# *Escherichia coli* is implicated in the development and manifestation of host susceptibility to the roundworm *Trichostrongylus colubriformis* infections in sheep

**DOI:** 10.1186/s13567-025-01565-1

**Published:** 2025-07-01

**Authors:** Fang Liu, Jody McNally, Damarius S. Fleming, Aaron B. Ingham, Peter William Hunt, Robert W. Li

**Affiliations:** 1 College of Public Health, Zhengzhou University, Zhengzhou, China; 2 CSIRO F.D. McMaster Laboratory, Armidale, NSW 2350 Australia; 3 Animal Parasitic Diseases Laboratory, USDA-ARS, Beltsville, MD USA; 4 CSIRO Agriculture and Food, St. Lucia, QLD 4067 Australia

**Keywords:** Breeding, *E. coli*, microbiome, nematodes, ovine, resistance, *Trichostrongylus*

## Abstract

Applied breeding for host resistance to gastrointestinal nematodes represents a cost-effective strategy for parasitic control. While resistance is under moderate genetic influences, gut microbial components involved in the development of resistance or susceptibility remain largely unknown. Here we characterize the structure and metabolic potential of the proximal colon microbiota in unique ovine populations bred for resistance and susceptibility using a full-length 16S rRNA gene sequencing-based microbiome approach. The resistant lambs produced significantly fewer parasite eggs than susceptible animals grazing on the same pasture. Further, the resistant lambs displayed a significant reduction in worm establishment in response to a *Trichostrongylus colubriformis* challenge infection (*P* < 0.0001; *N* = 20 per group). Among 32 bacterial species or strains displaying a significant difference in relative abundance between the resistant and susceptible group, *E. coli* was more abundant in susceptible lambs. *E. coli* was also ranked as the most important species in distinguishing the resistant and susceptible status. Moreover, a microbial signature or balance consisting of *E. coli* (Numerator) and *Parabacteroides distasonis* and *Bacteroides thetaiotaomicron* (Denominator) predicted the resistance status with high accuracy. The metagenome function prediction also revealed that several pathways related to infectious diseases, such as Shigellosis and pathogenic *E. coli* infection, were significantly altered between the two phenotypes. Our findings demonstrated that microbial signatures with a high predictive power for the resistance status can be developed as biomarkers to facilitate the selection for host resistance in sheep.

**Supplementary Information:**

The online version contains supplementary material available at 10.1186/s13567-025-01565-1.

## Introduction

The nematode parasites from the genus *Trichostrongylus* are ubiquitous in distribution and are of high relevance to food animal production [1, 2]. They can infect many herbivorous species, including cattle, small ruminants, rabbits, and wild ungulates [3]. According to the 2019 USDA National Animal Health Monitoring System Goat Study, the prevalence of *Trichostrongylus* species in all U.S. goat operations reached 97.2%, five times higher than that of *Haemonchus* in the same operations [4]. The geographical distribution of different *Trichostrongylus* species is generally defined by climate patterns. For example, *T. colubriformis* is prevalent in the summer rainfall zones of eastern Australia [5] and New Zealand [6], whereas *T. rugatus* is common in arid regions, such as the inland of Australia [7]. Further, each species often has its unique predilection site. *T. colubriformis* and *T. vitrinus* reside in the small intestine, while *T. axei* infects the abomasum. Moreover, some *Trichostrongylus* species, such as *T. colubriformis* and *T. axei*, are zoonotic and can infect humans [8, 9].

Rapid emergence and spread of anthelmintic resistance genes have markedly compromised the efficacy of existing anthelmintic drugs, a mainstay of parasite control strategies [10, 11]. As a result, breeding small ruminants for parasite resistance is increasingly appealing. A recent meta-analysis of 121 studies revealed that a global heritability estimate for host resistance to parasitic infections in sheep is moderate (*h*^*2*^ = 0.25) but stable, independent of breeds, age of assessment, and geographical locations [12]. Moreover, two widely used traits to estimate the degree of parasite infestation, worm egg counts (WEC) expressed in eggs per gram of feces and FAMACHA scores, a clinical evaluation system for anemia in small ruminants, are favorably correlated with performance traits, particularly growth, discounting concerns on a possible genetic trade-off between selection for resistance and performance [12]. As a result, more breeders, such as the US National Sheep Improvement Program (NSIP), have integrated the selection of parasite resistance in their applied breeding programs in small ruminants [13–17], which will play a critical role in sustainable ruminant production.

While multiple studies have recognized the effectiveness of using WEC estimated breeding value (EBV) in applied breeding in small ruminants [13, 18], WEC-based phenotyping has several issues. First, WEC is highly variable in response to genetics, seasonality, stages of animal husbandry, and management practices. A small percentage of animals, between 15 and 25%, even grazing on the same pasture, is responsible for the majority of parasite transmission, resulting in an over-dispersed distribution of WEC values [19]. This phenomenon not only presents some difficulties in model selections and data transformation but also requires a relatively large data pool. As a result, WEC measurements, while generally more accurate than other traits, such as body condition scores, are labor-intensive. FAMACHA is relatively easy to phenotype and tends to have a stronger correlation with production traits, but it is indicative of the status of anemia, only induced by blood-sucking parasites, such as *Haemonchus contortus* and *Bunostomum trigonocephalum* [20]. Moreover, several traits related to adaptive immunity, such as IgA and production of IL4 from stimulated whole blood, have been used as parasitological measures to predict WEC with some success in certain breeds [21]. However, their measurements can be cumbersome and do not appear to be applicable to all sheep breeds [22]. As a result, readily accessible biomarkers indicative of parasite burdens or predictive of parasite resistance are urgently needed to facilitate applied breeding.

The gut microbiota plays a critical role in ruminant physiology and animal health and well-being by modifying host–pathogen interactions. Early studies revealed that cattle partially immune to the stomach worm *Ostertagia ostertagi* possess the ability to maintain proper stability of their gut microbial ecosystem, contributing to the restoration of infection-impaired gastric function [23]. In goats, challenge infection by *H. contortus* altered the abundance of approximately 19% of the operational taxonomic units (OTU) and multiple metabolic pathways, including those relevant to butyrate biosynthesis and intestinal inflammation [24]. In sheep, infection by *H. contortus* also induced an alteration in gut microbiota [25]. Further, a high worm burden is associated with a relatively large change in gut microbial community composition, including significant differences in the relative abundances of *Firmicutes* and *Bacteroidetes* following infection [26]. The gut microbiota in small ruminants is also responsive to anthelmintic treatments [11]. While a single-dose moxidectin treatment of *H. contortus*-infected goats reduced both WEC and worm burden, it also significantly increased the abundance of *Proteobacteria*, particularly that of *Campylobacter*, in the proximal colon and affected several basic pathways, including bacterial secretion, butyrate metabolism, and LPS biosynthesis, and seemingly reduced the cellulolytic capacity in the colon [11]. A long-term anthelmintic treatment using controlled release capsules containing albendazole and abamectin significantly impacted the archaeal community in the sheep rumen [27]. *T. colubriformis* worms harbor a unique gut microbial community, distinguishable from that of their host. For example, *Mycoplasmoides* and *Stenotrophomonas* species are present in *T. colubriformis* worms but not in the duodenal microbiome of the ovine host [28]. These unique microbes may play a role in the survival of the parasite in the host. However, the mechanisms by which the gut microbiota shapes the host-parasite interactions are still lacking. The causal relations between the gut microbiota and the development of host resistance or susceptibility to parasitic infections remain elusive. In this study, we attempt to identify gut microbial biomarkers with high predictive accuracy for host resistance and susceptibility using a full-length 16S rRNA gene sequencing based microbiome approach by taking advantage of our unique resource sheep populations developed by decades’ selective breeding for resistance or susceptibility to *H. contortus* and *T. colubriformis* [29].

## Materials and methods

The animal study was conducted at the Commonwealth Scientific and Industrial Research Organisation (CSIRO) F.D. McMaster Laboratory in Armidale, Australia. All procedures were carefully reviewed, approved, and monitored by the CSIRO Armidale Animal Ethics Committee (Animal Research Authority Numbers# 10/22 and 08/23). The animal use protocol and procedures were in compliance with the Australian Code for the Care and Use of Animals for Scientific Purposes and all relevant Commonwealth, State and Territory legislation on animal welfare. The study utilized the selection flocks (*Haemonchus* selection flock, HSF or *Trichostrongylus* selection flock, TSF) in the Merino breed, which have been subject to divergent genetic selection since the mid-1970s. The selection has produced a parasite resistant and a parasite susceptible line in each flock which have been kept separate since the onset of selection [30]. Feces from 247 sheep, at six to eight months of age, in the two resistant and two susceptible CSIRO parasite selection lines were collected after these animals grazed as a contemporary group on the same pasture during their first grazing season. Fecal samples were taken from the rectum and analyzed immediately to measure WEC using the McMaster method. The resistant and susceptible lines were divergent in WEC values as expected. Animals were ranked using the WEC values and the ten animals with the lowest field WEC from each resistant line (TSF and HSF) and the ten animals with the highest field WEC from each susceptible line were chosen for a subsequent controlled infection study (for a total of 40 male lambs including 20 resistant and 20 susceptible animals). Over the 1^st^ and 2^nd^ days after their transfer from the pasture to the animal house pens, all animals were treated with multiple commercial drench products, such as Startect^®^, Rametin^™^, Zolvix^™^ Plus, WSD Closantel^™^, and Flukazole C, following label instructions to remove existing nematode, trematode and cestode parasites. The combined treatments delivered 0.04 mg naphthalophos, 0.60 mg abamectin, 2.00 mg derquantel, 2.50 mg monepantel, 3.75 mg praziquantel, 4.53 mg oxfendazole, 7.50 mg closantel, and 12.00 mg triclabendazole per kg of liveweight. The animals were also vaccinated using Websters 6-in-1 at a dose of 1 mL per head, to protect against clostridial diseases and *Corynebacterium* infections. These animals were housed in four group pens each containing animals from the four selection lines. Pens were supplied with water troughs and environmental enrichment. The animals were fed 700 g/day animal house pellets (lucerne based pellets) plus heat treated chaff. On the 13^th^ day of the 14 days acclimation period, feces were sampled from each animal and analyzed to detect any roundworm eggs; no animals had WEC values greater than zero. On the 14^th^ days after introduction to the animal house, each animal received 20 000 T*. colubriformis* infective larvae in a single dose administered orally. The infection was allowed to progress for 14 days; and animals were sacrificed prior to sexual maturity of the infecting parasites. As a result, WEC measurement from these lambs could not be undertaken. On the day before necropsy, the animals were weighed and not fed. On the 14^th^ day post-challenge infection, the animals were euthanized by the captive bolt stunning method followed by exsanguination. Contents of the proximal colon and rumen fluid were collected for total DNA extraction for microbiome studies. The contents of the first three meters of the jejunum were then collected for worm counts. Contents were collected, the jejunum section split, scraped (to detach the mucous layer), and washed with water; and the scrapings and wash water were then added to the contents. The combined contents, washing water and scrapings for each animal were brought to a total volume of 500 mL using water and were thoroughly mixed before taking two 30 mL subsamples which were frozen immediately at −20 °C to preserve them. Thawed samples were examined under low power (10–50 X) on a dissecting scope to count male, female and juvenile nematodes. Both aliquots were counted in total for each animal. All nematodes observed belong to *Trichostrongylus*.

### Full-length 16S rRNA gene sequencing-based microbiome analysis

Total DNA was extracted from proximal colon content and rumen fluid samples collected at necropsy using a QIAamp Fast DNA Stool Mini kit (Qiagen, Germantown, MD, USA) with modifications [31]. A bead-beating procedure using a Mini-Beadbeater-96 instrument (Biospec, Bartlesville, OK, USA) and Lysing Matrix E (MP Biologicals, Irvine, CA, USA) was performed. Lysis at 70 °C was extended to 8 min. Both nuclease free water as a non-DNA template control and the microbiome standard D6331 (Zymo Research, Irvine, CA, USA) were processed along with experimental samples following the same protocol and parameters to validate the workflow. The full length 16S rRNA gene was amplified and sequenced based on the LoopSeq synthetic long-read chemistry (Element BioSciences, San Diego, CA, USA) using an Illumina NovoSeq 6000 sequencer [32]. Raw reads were first trimmed using Trimmomatic (v0.39) and then de novo assembled into full-length 16S sequences using the SPAdes algorithm in the LoopSeq proprietary pipeline. The resultant full-length 16S rRNA gene sequences were analyzed using the DADA2 algorithm (v1.16) to generate amplicon sequence variant (ASV) feature tables [33]. Bacterial species and strains were annotated against the SILVA Small Subunit (SSU) rRNA Database (release v138) at a 97% similarity threshold over the entire inquiry length. Raw sequence data were deposited to the NCBI SRA database (SRA accession/BioProject: PRJNA1214723). The raw count data can be found in the Digital Commons Data [34].

### Bioinformatic and statistical analyses

The ASV counts were first filtered to remove the features with very low and then transformed using the centered log ratio (CLR) method. The bacterial species and strains with significantly different abundance between the experimental groups, resistant (RES) and susceptible (SUS), or between the two selection lines (TSF vs HSF), were detected using the edgeR (v4.0) method [35] with a significance threshold at false discovery rate (FDR)-adjusted *q* values ≤ 0.05. The machine learning tool Random Forest (v4.7) was used to detect the important features that distinguish between resistant and susceptible phenotypes. The Random Forest (RF) parameters used were as follows: the number of trees in the forest (ntree) was set to 500 and the number of features randomly sampled at each node in a tree (mtry) was 7. Microbial association networks at the species level were constructed and compared using NetCoMi (v1.1.0) [36]. A greedy stepwise algorithm, *selbal* (v07_2019), was used to select balances or microbial signatures with predictive power for response variables [37]. MaAsLin2, a comprehensive R package for efficiently determining multivariable association between phenotypes and microbial features, was also utilized in the association analysis [38]. In addition, the algorithm Phylogenetic Investigation of Communities by Reconstruction of Unobserved States (PICRUSt2) in the QIIME pipeline was used for the prediction of metagenome function [39]. GraPhlAn (v0.9) was used to generate circular representations of taxonomic and phylogenetic trees [40].

The R Stats package (R v4.3.0) was used for statistical analyses. Because WEC values are not normally distributed, a non-parametric Wilcoxon Rank-Sum test (Mann Whitney U test) was used for two-group statistical comparisons. The number was expressed as means ± SD, unless stated otherwise. FDR corrections were also applied to unadjusted *P* values, where a significance threshold was considered at FDR-adjusted *q* values ≤ 0.05.

## Results

### Host resistance compromised the establishment of *Trichostrongylus colubriformis*

**Figure 1:**
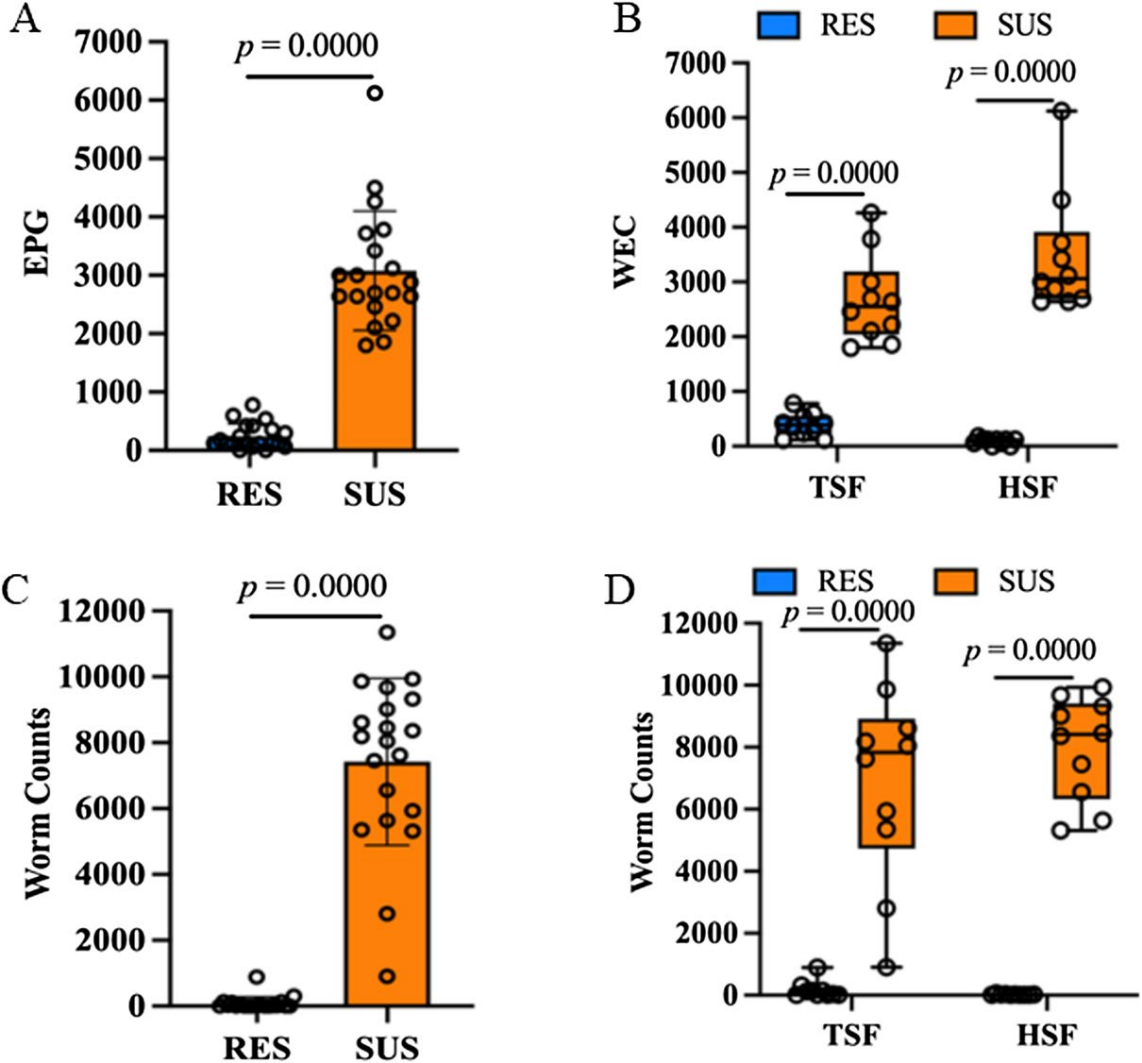
**Selective breeding compromised worm establishment in parasite resistance lambs.** WEC values represent the samples collected at a single time point while animals grazed on the pasture. **A**. WEC values in resistant (RES) and susceptible (SUS) animals grazing on the same pasture during their first grazing season. *N* = 20 per group. **B**. WEC values in RES and SUS phenotypes in each flock. TSF: flock targeting for *Trichostrongylus colubriformis*. HSF: flock targeting for *Haemonchus contortus* resistance. *N* = 10 per group. **C**. Worm counts between RES and SUS phenotypes regardless of selection flocks in response to a single dose *T. colubriformis* challenge infection. *N* = 20 per group. **D**. Worm counts between RES and SUS phenotypes in response to a single dose *T. colubriformis* challenge infection in each flock. *N* = 10 per group. Worm count values were obtained at necropsy at 14 days post-infection. The data are presented as Mean ± SD.

Parasitic egg shedding of 247 lambs, at six to eight months of age, grazing on the same pasture during their first grazing season was monitored by counting WEC. These lambs include 118 resistant and 129 susceptible animals in two flocks, HSF (149 lambs) and TSH (98 lambs), respectively. Overall, the resistant lambs had significantly lower WEC than the susceptible lambs while grazing on the pasture (*P* = 1.05 × 10^–20^; *N* = 247), regardless of the flock (Figure 1A). Within each flock, the results followed a similar trend: the resistant lambs had three times lower WEC values (*P* < 10^–10^; Figure 1B). For example, the WEC of susceptible lambs from the HSF flock was 3.37 fold higher than that of resistant lambs in the same flock, 1883.51 ± 1456.25 and 558.40 ± 498.25 (mean ± SD), respectively (*P* = 9.72 × 10^–12^). However, no significant differences between the two flocks were detected (data not shown). The WEC data from all 247 lambs with natural exposure were ranked; and 40 lambs, 20 from RES and 20 from SUS selection lines, were then recruited into the challenge infection experiment based on their relative rankings. The WEC data from these 40 lambs were similar to the overall trend, i.e., the resistant lambs produced significantly fewer parasite eggs than the susceptible animals while there were no statistically significant differences between the two flocks.

The development of resistance significantly impaired the parasite worm establishment. At 14 days post challenge infection (DPI) with 20 000 T*. colubriformis* infective larvae, the number of parasite worms recovered between the resistant and susceptible lambs were 87.92 ± 201.94 and 7421.25 ± 2522.96, respectively (Z-score = 5.3965; two-tailed Wilcoxon *P* value = 0.0000; *N* = 20 per group; Figure 1C), regardless of the flock. Within each flock, the resistant lambs also had significantly lower worm counts than the susceptible lambs (Figure 1D). Of note, selection appeared to non-specifically improve host resistance despite the parasite species that was originally targeted. For example, the animals which underwent decades’ selection for *T. colubriformis* resistance (TSF) performed equivalently with those selected for *H. contortus* resistance (HSF), in response to *T. colubriformis* challenge infection. This observation was consistent with our previous findings [29, 41].

### The lambs susceptible to *T. colubriformis* infection harbored a distinct microbial community

**Figure 2:**
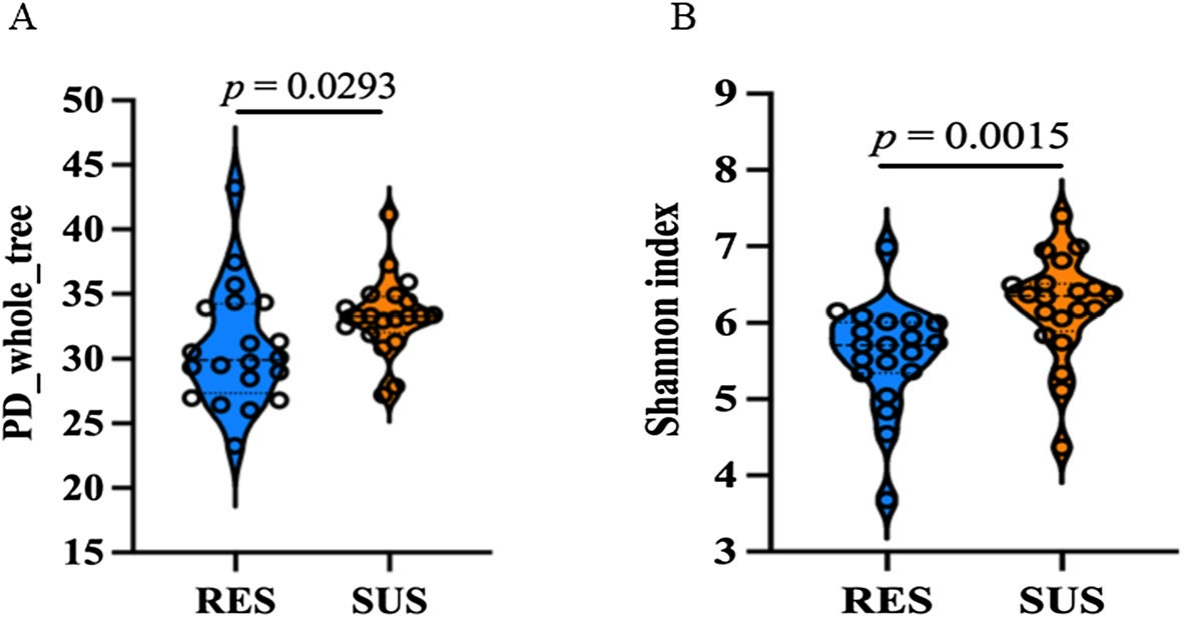
**The differences in alpha diversity between resistant and susceptible lambs in response to** ***Trichostrongylus colubriformis*** **infection**. RES: lambs bred for parasite resistance. SUS: lambs bred for parasite susceptibility. **A**. Phylogenetic diversity (PD_whole tree). **B**. Shannon index. The data were presented as Mean ± SD. The *P* value was calculated using two-tailed Wilcoxon rank-sum test. *N* = 20 per group.

The infection with the worms that reside in the jejunum seemingly had no measurable impact on the foregut microbiome. For example, the alpha diversity indices in the rumen between the resistant and susceptible lambs were indistinguishable despite the large difference in the intensity of infection. Nevertheless, the challenge infection resulted in a significant difference in alpha diversity in the proximal colon (PC) microbial community (Additional file 1). The richer phylogenetic diversity (PD whole tree) was evident in the PC microbiome of the susceptible (33.34 ± 3.00) compared with the resistant lambs (30.89 ± 4.60; *P* = 0.0293; *N* = 20 per group; Figure 2A). The Shannon index was also significantly higher in the susceptible lambs than in the resistant lambs in each flock (Figure 2B). However, the species richness index, such as Chao1, between the susceptible and resistant lambs was not different (*P* > 0.05). The beta diversity was assessed using Principal Coordinates Analysis (PCoA) as the method of ordination. The difference in the beta diversity index between the resistant and susceptible groups was also not statistically significant (*P* > 0.05; Additional file 2).

**Figure 3:**
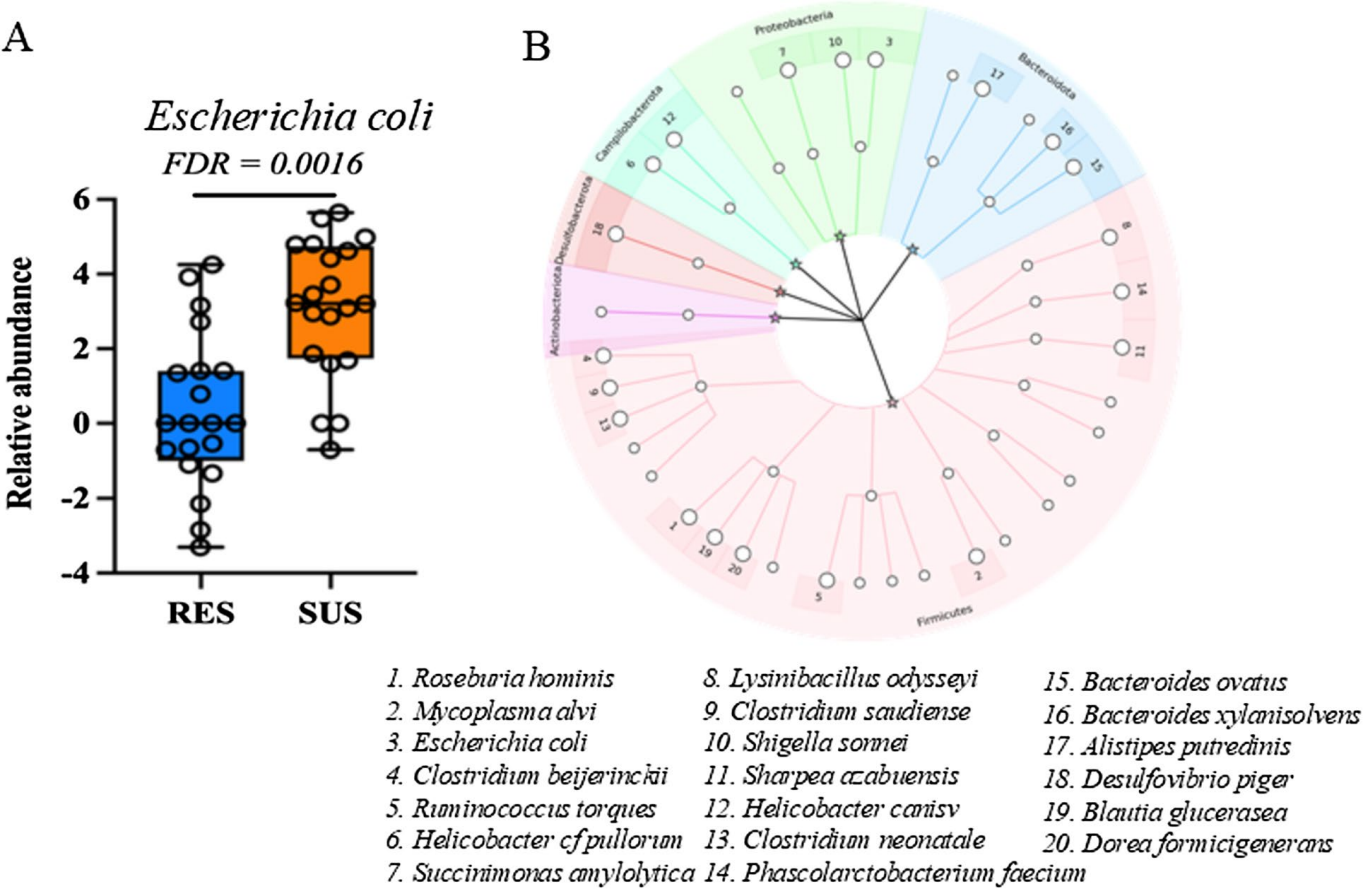
***E. coli*** **and other bacterial species differed significantly between two phenotypes in response to** ***Trichostrongylus colubriformis*** **infection**. RES: lambs bred for parasite resistance. SUS: lambs bred for parasite susceptibility. **A.** E. coli. **B**. The circular representations of taxonomic and phylogenetic trees affected by the infection in the two phenotypes. The numbers in the tip of the tree represent the one of the species with significant differences in relative abundance between resistant and susceptible phenotypes in response to infection at FDR < 0.05 as detected using the edgeR algorithm. The Y-axis in the bar charts denotes the relevant abundance. The data are presented as Mean ± SD. FDR: false discovery rate adjusted *P* values (q). *N* = 20 per group.

Thirty two species and strains (including at least 20 bacterial species) displayed significant differences in their relative abundance between the resistant and susceptible groups at FDR < 0.05, as detected using the edgeR algorithm (Additional file 3). For example, *Clostridium beijerinckii* and *C. neonatale* were greater than tenfold more abundant in the resistant group than the susceptible group. On the other hand, the abundance of *E. coli* (Figure 3A), as well as its three strains, KTE71, KTE103, and O104:H4_str_Ec11_9990, were significantly lower in the resistant than susceptible groups (Additional file 3). *Fibrobacter succinogenes*, one of the most predominant species in the proximal colon, did not show significant difference in the relative abundance between the resistant and susceptible groups at the species level. Nevertheless, one of its two subspecies, *F. succinogenes subsp. succinogenes S85*, was enriched in the resistant lambs (Additional file 3). Two species, *Helicobacter cf. pullorum* and *Ruminococcus torques*, which were more abundant in the resistant group, also displayed significant differences in relative abundance between the two flocks (TSF and HSF). For example, the abundance of *R. torques* was significantly higher in resistant than susceptible lambs, but also higher in the HSF group than in the TSF group (*P* = 0.0070 and 0.0021, respectively). At the genus level, eight genera, such as *Clostridium* and *Negativibacillus*, had significant differences in relative abundance between the resistant and susceptible phenotypes, whereas the abundance of five genera, including *Dorea*, *Desulfovibrio*, and *Negativibacillus*, differed significantly between the two flocks at FDR < 0.05. Three phyla, *Actinobacteria*, *Bacteroidota*, and *Firmicutes* differed significantly between the resistant and susceptible phenotypes (Figure 3B).

**Figure 4:**
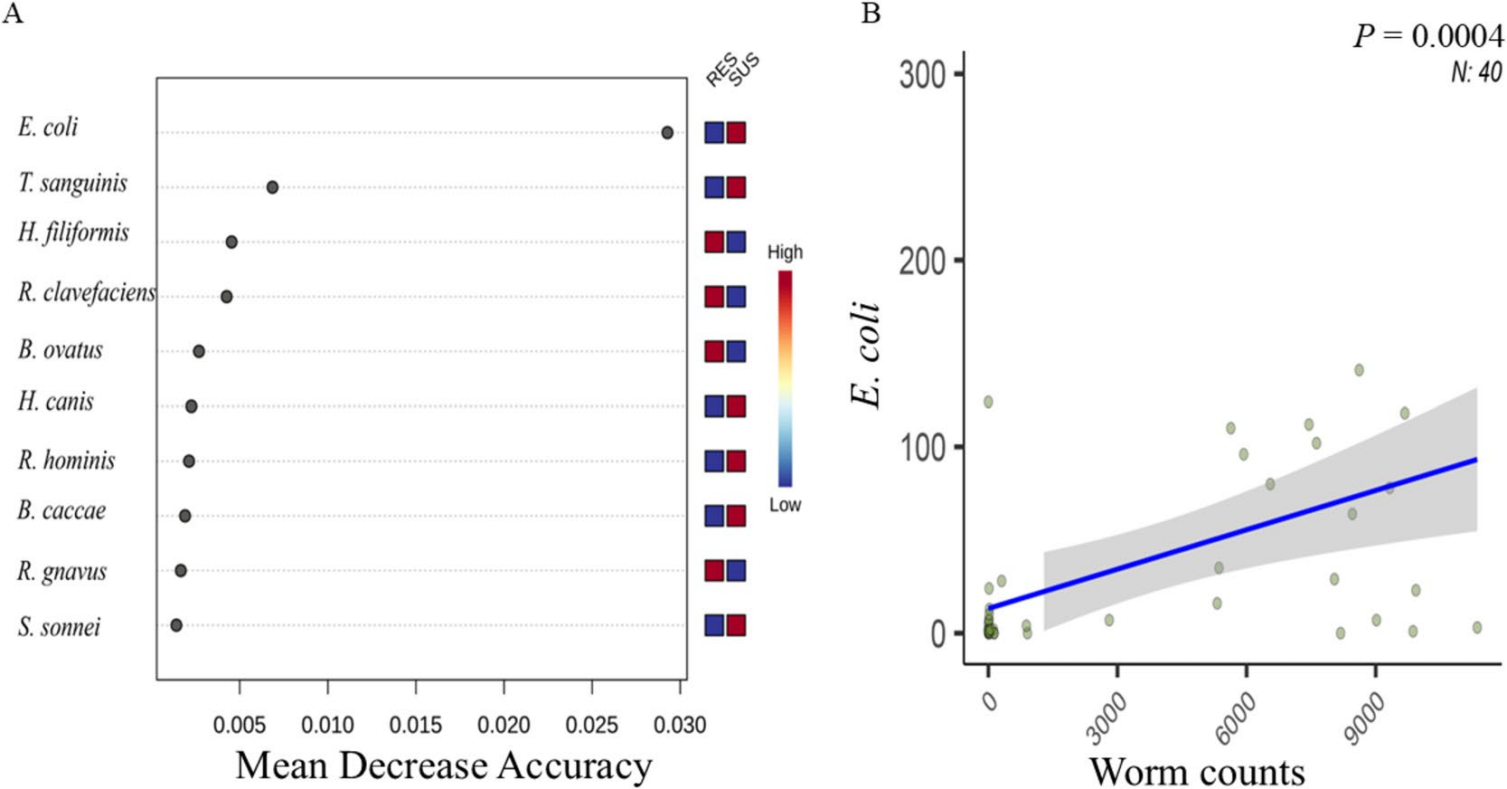
***E. coli*** **as the most important microbe in distinguishing parasite resistance status in sheep. A**. The Random Forest (RF) classification model identified the 10 most important species discriminative of the status of resistance status. The color bar denotes relative abundance (high vs low). **B**. The normalized *E. coli* abundance (count) was significantly correleted with worm burden as calcalued by the MaAsLin2 algorithm. Shade: 95% confidnece interval. *N* = 40.

**Figure 5:**
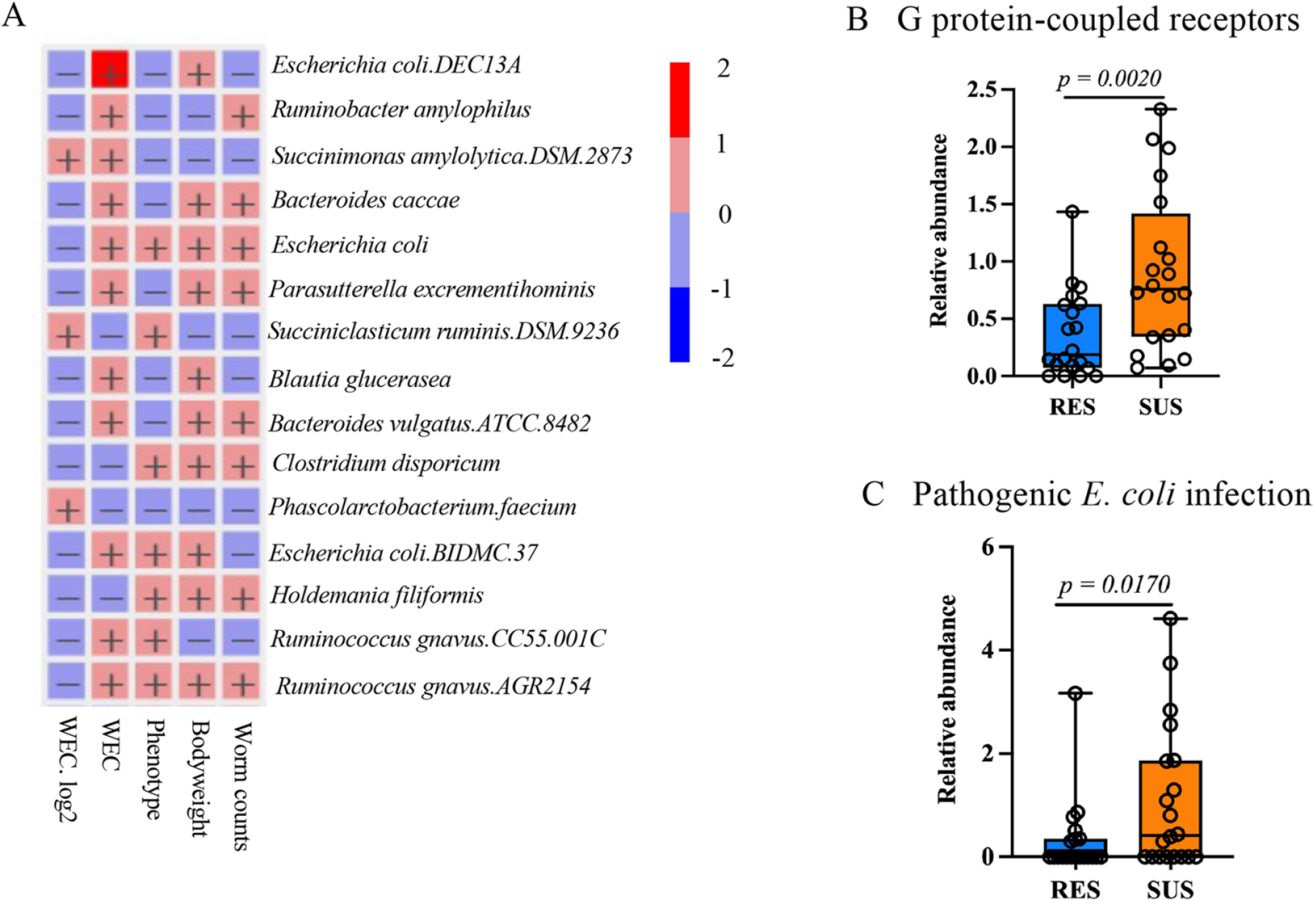
**Gut bacterial species correlated with parasitological traits and metagenome function.** **A**. Top 15 speceis or strains significantly correlated with five features, including WEC, log_2_WEC, worm counts, bodyweight, phenotype (resistance vs susceptibility). The + symbol denotes significance at *P* < 0.05. The color bar on the right indicates fold changes in relative abundance. **B**. Kyoto Encyclopedia of Genes and Genomes (KEGG) pathway category G-protein coupled receptors preidtced using the PICRUSt2 algorithm. C. The number of hits (relaytive abundance; Mean ± SD) annoated to the KEGG pathway termed Pathogenic *E. coli* infection estimated using PICRUSt2.

The RF classification model was used to rank variables that better distinguish the resistance status. As Figure 4A shows, *E. coli*, *Turicibacter sanguinis*, and *Holdemania filiformis* were the three most important species able to predict resistant and susceptible phenotypes based on their mean decrease accuracy. Intriguingly, *E. coli*, which was ~ five fold higher in relative abundance in the susceptible than resistant groups, was also the species significantly associated with worm count data (*P* = 0.0004; Figure 4B) and WEC value (*P* = 0.0016; Figure 5A). Two strains of *E. coli*, KTE34 and O104.H4.str. Ec11.9990, were also significantly associated with the worm count (*P* = 0.0025 and 0.0040, respectively). *Eubacterium hallii* strain DSM3353 was also among the few species/strains strongly associated with both WEC and log_2_ transformed WEC (*P* = 0.0021 and 0.0015, respectively).

The metagenome function between resistant and susceptible lambs was predicted using PICRUSt2 [39], which has markedly improved accuracy over the previously published methods by including over 41 000 fully sequenced bacterial and archaeal genomes. The 12 most abundant Kyoto Encyclopedia of Genes and Genomes (KEGG) pathways predicted from the gut microbiota of the resistant and susceptible lambs were identical, including transporters (as well as ABC transporters), DNA repair and recombination proteins, ribosome (and ribosome biogenesis), purine and pyrimidine metabolism, peptidases, chromosome, amino acid related enzymes, and the two-component system. At least four KEGG pathways were significantly enriched in the susceptible animals at a combined cutoff of two-tailed Wilcoxon *P* < 0.05 and twofold differences in predicted numbers of hits, including one involved in environmental information processing, G protein-coupled receptors related signaling and interactions (Figure 5B), and infectious diseases, such as Shigellosis and pathogenic *E. coli* infection (Figure 5C). Further examinations of our data against KEGG Orthology (KO) database identified 356 KO functional orthologs (proteins) at an unadjusted *P* value < 0.01. 2-succinyl-6-hydroxy-2,4-cyclohexadiene-1-carboxylate synthase (K08680), molybdenum transport protein (K03813), and drug/metabolite transporter, DME family (K03298) were all two fold more abundant in the susceptible than the resistant animals (Wilcoxon *P* < 0.0001). Phage shock protein (psp) B (K03970) was also five fold more abundant in the gut of the susceptible sheep (*P* < 0.01). This ortholog is part of a prokaryotic defense system and readily inducible by various stimuli, such as heat, ethanol, osmotic shock and phage infection, while the gene encoding this protein exists as a four-gene psb operon in *E. coli* genomes [42]. Among the few orthologs more abundant in the gut of the resistant sheep stands out histidine ammonia-lyase (K01745, histidase), an enzyme involved in histidine degradation and utilization in the gut.

**Figure 6:**
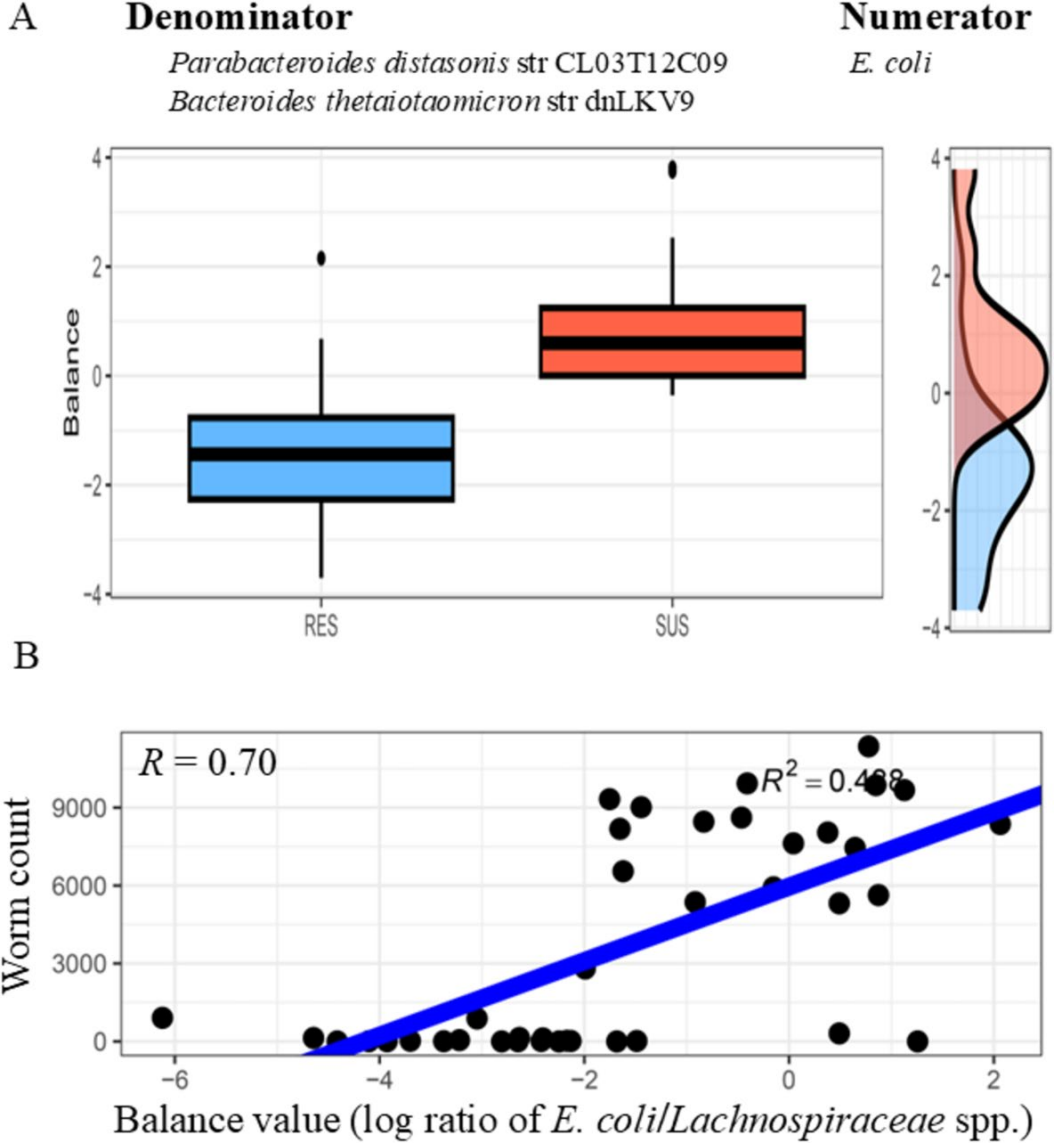
**Gut microbial signatures with predictive accuracy for parasite resistance.** **A**. The balance (or abundance log ratios) consisting of *E. coli* (numerator) and two bacterial strains, *Parasbacteroides distasonis* str CL03T12C09 and *Bacteroides thetaiotaomicron* str dnLKV9 (denominator) able to predict resistant and susceptible phenotypes with high accuracy (76%). The higher balance values (Y-axis) indicate the parasite susceptibility (SUS). RES: parasite resistance. **B**. A microbial signature consisting of *E. coli* (numerator) and a *Lachnospiraceae* bacterium (denominator) was significantly correlated with worm counts (R^2^ = 0.49 or R = 0.70).

Microbial balances (log ratios) consisting of *E. coli* (Numerator) and *Parabacteroides distasonis* strain CL03T12C09 and *Bacteroides thetaiotaomicron* strain dnLKV9 (Denominator) were able to predict the resistance status (RES vs SUS) with a mean accuracy of 76% (Figure 6A). The negative balance values were indicative of the resistant phenotype. Another balance, consisting of *R. flavefaciens* FD-1 (Numerator) and *B. thetaiotaomicron* strain dnLKV9 and *Lactobacillus mucosae* strain LM1 (Denominator) were predictive of the flock (mean accuracy 72%). TSF flock samples tended to have higher balance values than HSF flock samples, regardless of the RES or SUS status. Moreover, the two balances, consisting of *E. coli* (Numerator) and a *Lachnospiraceae* bacterium (Denominator) and *Eubacterium hallii* strain DSM3353 (Numerator) and *B. vulgatus* strain CL09T03C04 (Denominator) were weakly correlated with worm counts and log^2^ WEC values, respectively (R^2^ = 0.49, Figure 6B and 0.39, respectively).

### The resistant lambs had a distinct pattern of gut microbial interactions

**Figure 7:**
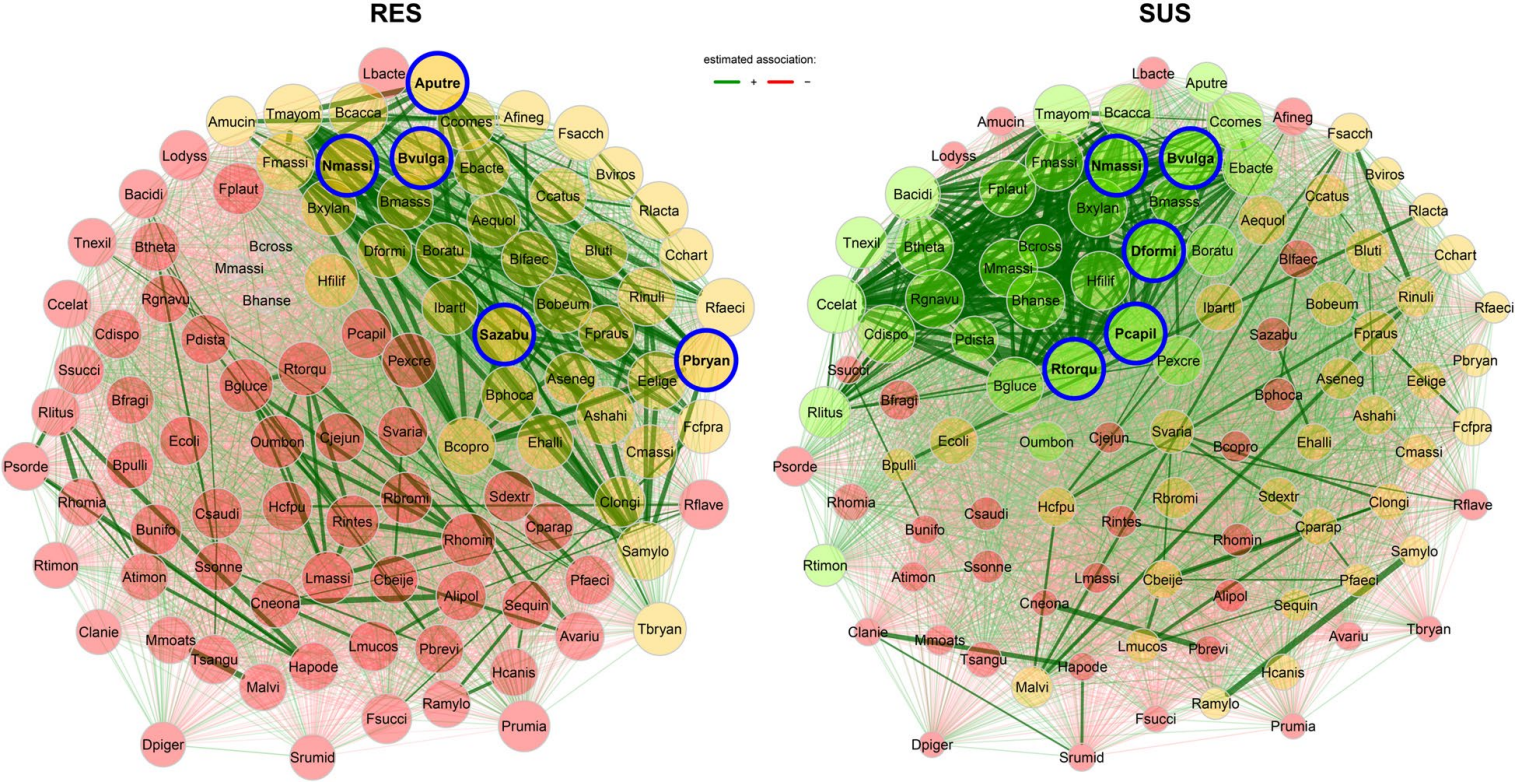
**Bacterial interaction networks between parasite resistant and susceptible lambs in response to** ***Trichostrongylus colubriformis*** **infection**. The interaction or association networks were constructed using NetCoMi. Network hubs were highlighted in a bold font with borders. The edge color represents the positive (Green) or negative (Red) interactions, as determined using the greedy modularity optimization procedure. The color of nodes represents the module, a cluster of functionally relevant species. The width of the edge denotes the strength of each interaction. Species symbols were abbreviated using the 1^st^ capital letter for the genus name followed by four or 5 letters representing species names. The full taxon names in the networks can be found in Additional file 5. RES: parasite resistant lambs. SUS: parasite susceptible lambs. *N* = 20 per group.

Bacterial interactions at a species level between the resistant and susceptible lambs were compared using NetCoMi. The global network properties between the two phenotypes were highly similar. For example, clustering coefficient, edge density and connectivity, and average path length in the networks derived from these two phenotypes were close. Each network had three modules or clusters (Figure 7). Each network had five hub nodes. In the network from the resistant lambs, the hub nodes included *Alistipes putredinis*, *Prevotella bryantii*, and *Sharpea azabuensis*, whereas *Dorea formicigenerans*, *Pseudoflavonifractor capillosus*, and *R. torques* were among the hub nodes in the susceptible network. *Bacteroides vulgatus* and *Negativibacillus massiliensis* acted as hub nodes in both networks. As a hub species in the resistant network, *A. putredinis* is positively correlated with at least seven other species with correlation coefficient values *R* > 0.97, including *A. shahii*, *B. coprocola*, *E. eligens*, *R. inulinivorans*, and *Succinimonas amylolytica*. One of the other hub nodes, *P. bryantii*, was also strongly correlated with some of these species, such as *Eubacterium eligens*, *R. inulinivorans*, and *S. amylolytica*. On the other hand, *B. vulgatus* as a hub was negatively correlated with *Desulfovibrio piger* (R = −0.54)*.* In the susceptible network, *D. formicigenerans* as a hub node was strongly associated with other hub nodes, such as *Pseudoflavonifractor capillosus* and *N. massiliensis* (*R* > 0.97). Further, *R. torques* as a hub node was positively associated with *Blautia hansenii*, *C. celatum*, *C. disporicum*, *H. filiformis*, and *Terrisporobacter mayombei* at a stringent cutoff *R* > 0.99 in the susceptible network. Notably, there were several species showing differential associations in the two networks, i.e., positive association in one network but negative association with the same node in another network. For example, *R. torques* was strongly and positively correlated with both *B. hansenii* and *C. disporicum* in the susceptible network (*R* > 0.994) but not correlated or negatively correlated with the same two nodes in the resistant network (Additional file 4). The abbreviations for species names in the networks can be found in Additional file 5.

## Discussion

The role of the gut microbiota in modifying host-parasite relations has been only recently appreciated. Host-microbiota-parasite interactions are complicated and reciprocal; and myriads of host, parasite, and environmental factors are likely involved. While causal effects remain elusive, both direct and indirect actions are likely in play [43]. Multiple gastrointestinal (GI) nematodes, such as *H. contortus* and *T. colubriformis*, penetrate host tissues, at least partially during their life cycles, which alter gut microenvironment and intestinal permeability. For example, during *H. contortus* primary infection in goats, the luminal pH values in the abomasum are significantly increased, allowing certain bacteria to flourish [24]. While residing in the gut mucosa or lumen, many nematodes also compete with the host for nutrients, resulting in nutrient deficiency for the host and compromising normal host physiology. Indirect actions of parasites on the gut microbiota can be revealed by studying the microbiome before and after anthelmintic treatment [11, 27]. Aside from the direct effect of parasites, another critical interface in host-microbiota-parasite interactions is the host immune system. The infection-induced changes in the gut microbiota have significant impacts on the host immunity, altering susceptibility to subsequent viral and bacterial infections [44], likely via pathogen-associated molecular patterns (PAMPs), such as lipopolysaccharides and peptidoglycans, from gut microbes and bacterial metabolites, such as short-chain fatty acids, antimicrobial peptides and bactericidal, and other signaling molecules in fatty acid metabolism and ethanolamine utilization [43, 45]. For example, anti-inflammatory treatments using corticosteroids can alleviate the symptom of *Teladorsagia circumcincta* infection in lambs [46]. However, much of our knowledge on intricate interplays among the host, microbiota, and parasites is derived from rodent models. Mechanistic studies attempting to dissect intimate interactions among the host, gut microbiota, and parasites using ruminant models are still lacking.

The studies using model nematodes (such as *Caenorhabditis elegans*) or parasites of rodent hosts (such as *Trichuris muris*) demonstrate that parasites require bacteria for their development and survival [47]. Using an in vitro culture system, it was demonstrated that *T. muris* egg hatching requires the presence of *E. coli* [48]; and notably this hatching occurs within the GI tract, unlike the lifecycle of *T. colubriformis*. By controlling numbers of the bacteria in the host, the number of hatched *T. muris* eggs can be significantly reduced. Moreover, *E. coli*-dependent egg hatching requires its microbial byproducts, such as those in fatty acid biosynthesis and ethanolamine utilization pathways [45] as well as proteases of bacterial or parasite origin or chitinases that worms secret, which aid the degradation of the egg polar plugs that leads to larval eclosion [49]. Other bacterial species, such as those from the family *Peptostreptococcaceae*, have superior capacity to promote the life cycle of *Trichuris* species, including hatching of eggs from *T. muris* and *T. trichiura* [50]. However, the bacterial requirements during the life cycle of GI nematodes, including egg hatching, exsheathment and establishment in the GI tract, in ruminants have yet to be studied. Nevertheless, our findings show that *E. coli*, including three *E. coli* strains or isolates, KTE71, KTE103, and Ec11_9990, were fivefold more abundant in the proximal colon of the susceptible than the resistant lamb. *E. coli* was significantly correlated with worm burden (*P* < 0.01). The balance consisting of *E. coli* (numerator) and an uncharacterized strain TF01.11 in the family *Lachnospiraceae* (denominator) was correlated with worm burden (R^2^ = 0.49) while another balance, consisting of *E. coli* (Numerator) and *P. distasonis* strain CL03T12C09 and *B. thetaiotaomicron* strain dnLKV9 (Denominator), was able to predict the resistant and susceptible phenotypes with high accuracy. If validated, this balance could be developed into a readily accessible microbial biomarker, overcoming many shortcomings of the WEC parameters commonly used during applied breeding. Future work will attempt to define the bacterial requirement of *T. colubriformis* egg hatching at species or strain levels. If *E. coli* is indeed required for egg hatching or larval development of GI nematodes in ruminants, as demonstrated in the whipworm system, we could develop a phage therapy specifically eliminating *E. coli* and its critical strains as a means for efficacious parasite controls in small ruminants.

The metagenome function prediction algorithm used in our study uncovered that protein hits assigned to two KEGG pathways, G protein-coupled receptors (Figure 5B) and pathogenic *Escherichia coli* infection (Figure 5C), were significantly enriched in susceptible lambs. G protein-coupled receptors (GPCR) play vital roles in both host and parasite functions and are essential to host immunity and helminth reproduction and muscle contraction [51, 52]. A recent study demonstrated that the anthelmintic praziquantel, which was one of the dewormers used to remove existing parasites in this study, is a serotoninergic G protein-coupled receptor ligand in humans [53]. Praziquantel likely interacts with both parasite and host GPCR receptors, contributing to its clinical efficacy by combining a deleterious paralytic effect on the parasite while promoting worm clearance in the host [53]. Additional data on the presence of such a specific receptor in our dataset and its relative abundance are still needed. Nevertheless, our findings provide motivation for future investigations in searching for effective parasite control strategies. On the other hand, the hallmark of infections by *E. coli* pathogenic strains, such as enteropathogenic *E. coli* (EPEC) and enterohemorrhagic *E. coli* (EHEC), is the induction of attaching and effacing lesions that damage intestinal epithelial cells, compromising normal physiology and nutrient absorption of hosts. An array of bacterial proteins or enterotoxins secreted by the Type III secretion system interact with host receptors. These virulence factors can also hijack global gene expression as well as post-translational mechanisms, such as protein phosphorylation, in the host [54]. In ruminants, diarrhea caused by pathogenic *E. coli* is a major cause of death with a mortality rate of over 50%. Challenge infections by pathogenic *E. coli* strain O1 result in bacterial dysbiosis and reduces biosynthesis of short-chain fatty acid in calves [55]. Research attempting to understand the pathophysiological relevance of these important pathways in sheep is warranted.

The rumen and hindgut of ruminants harbor many cellulose-degrading bacteria, such as *F. succinogenes*, *R. albus* and *R. flavefaciens*. These species as well as those yet to be cultivated are responsible for digestion and utilization of lignocellulosic materials by ruminants to convert plant fibers to meat, dairy and wool products for human consumption. In our study, six *Ruminococcus* species (20 strains), such as *R. albus*, *R. bromii*, *R. flavefaciens*, and *R. torques*, were detected. In addition, *F. succinogenes*, the second most abundant species in the ovine proximal colon in our study, accounted for 9.15% of all species/strains identified. Both subspecies of *F. succinogenes* (subsp. *succinogenes* and subsp. *elongata*), were abundant in the ovine proximal colon microbiota. Intriguingly, *F. succinogenes* subsp. *succinogenes* was ~ seven times more abundant in the proximal colon microbiota of the resistant animals than the susceptible group (FDR = 0.0017). In pure culture systems, previous findings demonstrated that *F. succinogenes* is able to degrade a greater amount of cellulose from intact forage than the two other predominant cellulolytic bacteria, *R. albus* and *R. flavefaciens*, suggesting that *F. succinogenes* is a highly effective cellulose degrader [56]. Better cellulose degradation and utilization are the key to animal growth in organic ruminant production systems. Our data showed that the resistant sheep also harbored abundant *F. succinogenes* subsp. *succinogenes*; and a mechanistic understanding of this correlation may be important for unraveling the relationship between parasitism and productivity in sheep.

Several published studies have recently examined gut microbial composition associated with host resistance in sheep [26, 57–59]. In a study in sheep in response to *H. contortus* experimental infection, the investigators compared the fecal microbial composition between pre- and post-infection and found that sheep with high parasite burden tended to display a large change in microbial community composition, including significant differences in the relative abundances of *Firmicutes* and *Bacteroidete*s following infection [26]. A recent study aimed to elucidate parasite-host-microbiota interactions and resistance to *H. contortus* in sheep [57]. Paz et al. (2022) compared the microbial composition of various gut segments of 10 highly helminth-susceptible (High-WEC) and 10 highly helminth-resistant (Low-WEC) sheep and found that four genera *Butyrivibrio*, *Mycoplasma*, *Lachnoclostridium* and *Succiniclasticum* differed significantly between the High-WEC and Low-WEC sheep, after taking all samples into account and adjusting for gut segments [58]. In Churra sheep infected with *Teladorsagia circumcincta*, resistant sheep had a higher number of butyrate-fermenting *Clostridium* and *Turicibacter* in abomasal contents [59]. We also found that butyrate-producing bacteria are involved in the development of resistance to *T. colubriformis* infection. However, our present study suffers some limitations. Due to constraints in physical and financial resources, we were unable to include uninfected control groups from each of the selection lines for predictive modeling. In addition, the lack of longitudinal sampling at various post-infection time points, particularly at a later stage of *T. colubriformis* direct life cycle when eggs are produced, was undesirable, because WEC values from the challenge infection would be of value for association studies. Nevertheless, our study is unique in several aspects. First, the resource populations we used have undergone decades of selective breeding; and resistant and susceptible lines are well differentiated and stable [29]. These animals displayed significant differences in both WEC and worm burden in settings with either natural (field) or controlled challenge infections. Second, we are among the first to study the response of the gut microbiota of these animals to a controlled *T. colubriformis* infection. Previous studies focused on *H. contortus* and *T. circumcincta* which are abomasal parasites, unlike *T. colubriformis* which resides in the jejunum. Despite being one of the most prevalent parasites infecting small ruminants globally, *T. colubriformis* has not received the same level of scientific attention as other important species. Third, we used a full-length 16S rRNA gene sequencing-based approach, which allows us to have higher resolution at bacterial species and strain level based on sequencing homology searching in the SILVA high quality ribosomal RNA database, unlike previously published studies using V3-V4 hypervariable regions that can only annotate bacterial taxa to a genus level. In addition, we applied advanced algorithms, including variable selection, machine learning, and species interaction inference in our study. For example, *selbal*, a forward selection method for the identification of bacterial species or strains whose balances (log ratios) are most associated with or predictive of the response variable (resistance status) was used in our study. These algorithms have proven powerful in detecting previously unrecognized microbial features implicated in the manifestation of host resistance. Our future work will focus on the mechanistic understanding of the observed changes in gut microbial features during parasite pathogenesis, particularly during the initiation stage of infection. Microbial biomarkers identified will need to be validated in multiple, large and independent populations to assess their utility for practical breeding. Nevertheless, our findings are also valuable for future research aiming to understand non-genetic influences of host resistance to intestinal parasites in ruminants.

## Supplementary Information

**Additional file 1.** **Raw values of alpha diversity indices in the microbiome of resistance and susceptible lambs. **The raw values of select alpha diversity indies, phylogenetic diversity (PD) whole tree, Chao1, and Shannon, for individual lambs were provided. RES: Resistant; SUS: Susceptible.

**Additional file 2. Beta diversity in the proximal colon microbiota of resistant (RES) and susceptible (SUS) lambs**. The beta diversity was assessed using Principal Coordinates Analysis (PCoA) as the method of ordination. The difference in the beta diversity index between the resistant and susceptible groups was also not statistically significant (*P* > 0.05). *N* = 20 per group.

**Additional file 3.Bacterial species or strains with significant differences in relative abundance between resistant and susceptible lambs.** The comparison was conducted using the edgeR algorithm, as implemented in the MicrobiomeAnalyst pipeline, at a false discovery rate (FDR) cutoff ≤ 0.05 . The raw counts were filtered and then normalized based on the centered log ratio method. CPM: counts per million. FC = fold changes (SUS/RES). N = 20 per group.

**Additional file 4.Differential bacterial associations or interactions inferred using the NetCoMi algorithm**. The sign + means positive interactions. The sign – means negative interactions. The thickness of the edge (interaction line) indicates the strength of correlations. RES: the interaction or association network inferred from the resistant lambs. SUS: the association network inferred from susceptible lambs. The full species name can be found in Additional file 4. *N* = 20 per group.

**Additional file 5.** **Species abbreviations used for network inference and comparison.**

## Data Availability

The datasets generated and/or analyzed during the current study are available in the NCBI SRA repository (SRA accession# PRJNA1214723). All other supporting materials can be found in the online supplementary materials as well as from the Mendeley Data [34] and are available from the corresponding author upon request.
