# Correction: *Escherichia coli* is implicated in the development and manifestation of host susceptibility to the roundworm *Trichostrongylus colubriformis* infections in sheep

**DOI:** 10.1186/s13567-025-01604-x

**Published:** 2025-10-14

**Authors:** Fang Liu, Jody McNally, Damarius S. Fleming, Aaron B. Ingham, Peter William Hunt, Robert W. Li

**Affiliations:** 1 College of Public Health, Zhengzhou University, Zhengzhou, China; 2 CSIRO F.D. McMaster Laboratory, Armidale, NSW 2350 Australia; 3 Animal Parasitic Diseases Laboratory, USDA-ARS, Beltsville, MD USA; 4 CSIRO Agriculture and Food, St. Lucia, QLD 4067 Australia

**Correction: Veterinary Research (2025) 56:133** 10.1186/s13567-025-01565-1

Following publication of the original article [1], the authors identified a spelling error in the name of the author Damarius S. Fleming.

The incorrect author names is: Damarius Flemming

The correct author names is: Damarius S. Fleming

The original article has been corrected.
