# Supplementary material for: Escherichia coli is implicated in the development and manifestation of host susceptibility to the roundworm Trichostrongylus colubriformis infections in sheep
Source: Vet Res. 2025 Jul 1;56:133. doi: 10.1186/s13567-025-01565-1 (PMC12220768; doi:10.1186/s13567-025-01565-1)
Supplement: Supplementary file 1 — Additional file 1. Raw values of alpha diversity indices in the microbiome of resistance and susceptible lambs. The raw values of select alpha diversity indies, phylogenetic diversity (PD) whole tree, Chao1, and Shannon, for individual lambs were provided. RES: Resistant; SUS: Susceptible. [file 13567_2025_1565_MOESM1_ESM.docx]

**Additional file 1.** **Raw values of alpha diversity indices in the microbiome of resistance and susceptible lambs.** The raw values of select alpha diversity indies, phylogenetic diversity (PD) whole tree, Chao1, and Shannon, for individual lambs were provided. RES: Resistant; SUS: Susceptible.

| Sample ID | Phenotype | Flock | PD_whole_tree | Shannon | Chao1 | Gut segment |
| --- | --- | --- | --- | --- | --- | --- |
| C6226B12 | RES | HSF | 28.9887 | 5.0390 | 715.5161 | Proximal colon |
| C6230A10 | RES | HSF | 23.2666 | 4.8358 | 548.0000 | Proximal colon |
| C6234A11 | RES | HSF | 35.7026 | 6.0143 | 897.0120 | Proximal colon |
| C6236B13 | RES | HSF | 29.5064 | 5.3610 | 967.0167 | Proximal colon |
| C6239A12 | RES | HSF | 31.1880 | 5.9916 | 835.1719 | Proximal colon |
| C6243A13 | RES | HSF | 33.9176 | 6.0821 | 1023.1139 | Proximal colon |
| C6247A14 | RES | HSF | 29.3590 | 5.5209 | 977.4444 | Proximal colon |
| C6250B14 | RES | HSF | 26.9678 | 4.5412 | 689.2542 | Proximal colon |
| C6251A15 | RES | HSF | 30.5356 | 5.7403 | 828.2963 | Proximal colon |
| C6253A16 | RES | HSF | 26.8066 | 5.8138 | 996.0492 | Proximal colon |
| C6176B01 | RES | TSF | 34.3806 | 6.0271 | 767.0685 | Proximal colon |
| C6177A01 | RES | TSF | 30.0836 | 5.3396 | 792.2679 | Proximal colon |
| C6179A02 | RES | TSF | 28.4796 | 5.7105 | 665.6250 | Proximal colon |
| C6181A03 | RES | TSF | 26.0473 | 5.6134 | 646.6935 | Proximal colon |
| C6182B02 | RES | TSF | 29.7444 | 5.8869 | 935.8654 | Proximal colon |
| C6183B03 | RES | TSF | 31.3450 | 5.7074 | 693.6667 | Proximal colon |
| C6184B04 | RES | TSF | 26.4428 | 3.6778 | 694.3443 | Proximal colon |
| C6186A04 | RES | TSF | 43.2324 | 6.9851 | 1270.4811 | Proximal colon |
| C6191B05 | RES | TSF | 37.4464 | 6.1475 | 1033.7051 | Proximal colon |
| C6192A05 | RES | TSF | 34.3465 | 5.4853 | 809.5000 | Proximal colon |
| C6194A06 | SUS | TSF | 33.3787 | 6.9483 | 934.0370 | Proximal colon |
| C6195B06 | SUS | TSF | 33.2977 | 6.1712 | 895.2500 | Proximal colon |
| C6196B07 | SUS | TSF | 34.9528 | 6.0598 | 881.5484 | Proximal colon |
| C6199A07 | SUS | TSF | 33.3159 | 6.3702 | 1053.6667 | Proximal colon |
| C6201A08 | SUS | TSF | 34.9009 | 6.4472 | 942.5909 | Proximal colon |
| C6202B08 | SUS | TSF | 35.9413 | 6.4055 | 944.7692 | Proximal colon |
| C6206A09 | SUS | TSF | 33.2642 | 6.8129 | 820.3457 | Proximal colon |
| C6207B09 | SUS | TSF | 31.3126 | 5.3329 | 883.8103 | Proximal colon |
| C6215B10 | SUS | TSF | 41.1574 | 7.3994 | 1094.4138 | Proximal colon |
| C6216B11 | SUS | TSF | 33.0747 | 6.3323 | 880.6351 | Proximal colon |
| C6256A17 | SUS | HSF | 27.8826 | 4.3671 | 617.4688 | Proximal colon |
| C6258A18 | SUS | HSF | 30.8512 | 5.1188 | 854.2794 | Proximal colon |
| C6260B15 | SUS | HSF | 27.2104 | 5.7436 | 972.9241 | Proximal colon |
| C6271A19 | SUS | HSF | 32.9493 | 6.5139 | 990.7206 | Proximal colon |
| C6274B16 | SUS | HSF | 37.2731 | 6.9876 | 879.2800 | Proximal colon |
| C6282B17 | SUS | HSF | 31.8909 | 6.4928 | 873.0441 | Proximal colon |
| C6287B18 | SUS | HSF | 33.9033 | 6.1378 | 975.3971 | Proximal colon |
| C6290B19 | SUS | HSF | 33.2425 | 6.3662 | 985.3875 | Proximal colon |
| C6296A20 | SUS | HSF | 32.5127 | 5.8392 | 808.2877 | Proximal colon |
| C6300B20 | SUS | HSF | 34.4464 | 6.1879 | 938.2593 | Proximal colon |
