# Supplementary material for: Escherichia coli is implicated in the development and manifestation of host susceptibility to the roundworm Trichostrongylus colubriformis infections in sheep
Source: Vet Res. 2025 Jul 1;56:133. doi: 10.1186/s13567-025-01565-1 (PMC12220768; doi:10.1186/s13567-025-01565-1)

**Additional file 2**. **Beta diversity in the proximal colon microbiota of resistant (RES) and susceptible (SUS) lambs.** The beta diversity was assessed using Principal Coordinates Analysis (PCoA) as the method of ordination. The difference in the beta diversity index between the resistant and susceptible groups was also not statistically significant (*P* > 0.05). *N* = 20 per group.


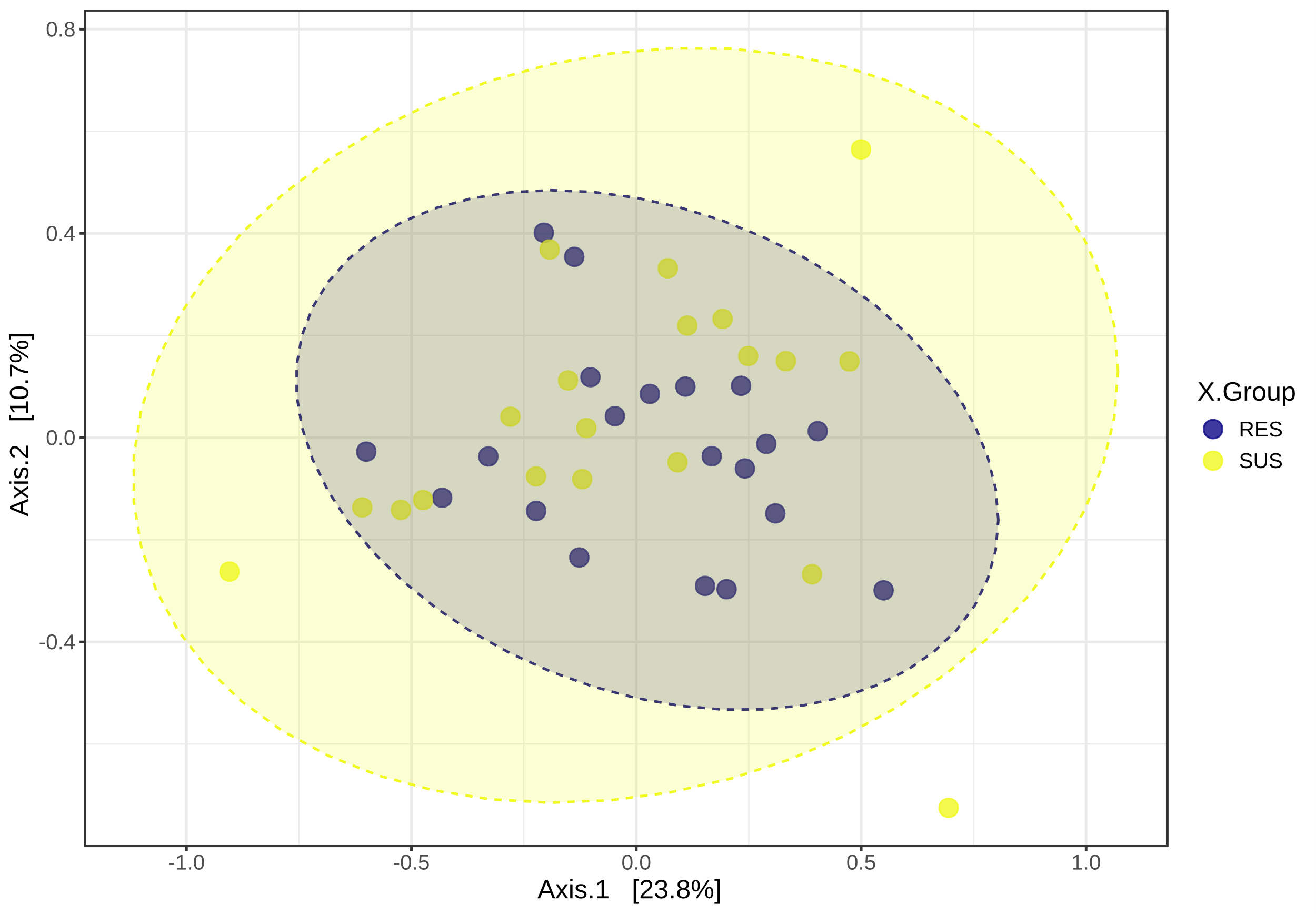

Supplement: Supplementary file 2 — Additional file 2. Beta diversity in the proximal colon microbiota of resistant (RES) and susceptible (SUS) lambs. The beta diversity was assessed using Principal Coordinates Analysis (PCoA) as the method of ordination. The difference in the beta diversity index between the resistant and susceptible groups was also not statistically significant (P > 0.05). N = 20 per group. [file 13567_2025_1565_MOESM2_ESM.docx]
