# Supplementary material for: Escherichia coli is implicated in the development and manifestation of host susceptibility to the roundworm Trichostrongylus colubriformis infections in sheep
Source: Vet Res. 2025 Jul 1;56:133. doi: 10.1186/s13567-025-01565-1 (PMC12220768; doi:10.1186/s13567-025-01565-1)
Supplement: Supplementary file 3 — Additional file 3.Bacterial species or strains with significant differences in relative abundance between resistant and susceptible lambs. The comparison was conducted using the edgeR algorithm, as implemented in the MicrobiomeAnalyst pipeline, at a false discovery rate (FDR) cutoff ≤ 0.05 . The raw counts were filtered and then normalized based on the centered log ratio method. CPM: counts per million. FC = fold changes (SUS/RES). N = 20 per group. [file 13567_2025_1565_MOESM3_ESM.docx]

**Additional file 3. Bacterial species or strains with significant differences in relative abundance between resistant and susceptible lambs**. The comparison was conducted using the edgeR algorithm, as implemented in the MicrobiomeAnalyst pipeline, at a false discovery rate (FDR) cutoff ≤ 0.05 . The raw counts were filtered and then normalized based on the centered log ratio method. CPM: counts per million. FC = fold changes (SUS/RES). *N* = 20 per group.

| **Species/Strain** | **log2FC** | **logCPM** | ***P* value** | **FDR** |
| --- | --- | --- | --- | --- |
| *Adlercreutzia equolifaciens* | -1.1781 | 8.1664 | 0.0105 | 0.0431 |
| *Alistipes putredinis DSM 17216* | -1.2328 | 7.7308 | 0.0029 | 0.0185 |
| *Alistipes timonensis JC136* | 1.4666 | 7.7961 | 0.0084 | 0.0360 |
| *Bacteroides fragilis CL03T12C07* | 2.1221 | 10.1260 | 0.0063 | 0.0310 |
| *Bacteroides fragilis str 3976T8* | 1.7792 | 8.8495 | 0.0085 | 0.0360 |
| *Bacteroides ovatus* | -2.5177 | 8.0233 | 1.37e-05 | 0.0006 |
| *Bacteroides ovatus CL02T12C04* | -1.4581 | 7.5256 | 0.0022 | 0.0168 |
| *Bacteroides ovatus CL03T12C18* | -1.4725 | 8.5259 | 0.0060 | 0.0303 |
| *Bacteroides vulgatus CL09T03C04* | -2.2951 | 13.2530 | 0.0016 | 0.0149 |
| *Bacteroides xylanisolvens CL03T12C04* | -1.7726 | 10.1680 | 0.0042 | 0.0232 |
| *Clostridium beijerinckii* | -4.3695 | 10.8010 | 1.30e-06 | 0.0002 |
| *Clostridium neonatale* | -3.3116 | 9.0011 | 3.37e-05 | 0.0009 |
| *Clostridium saudiense* | 1.4142 | 8.4933 | 0.0045 | 0.0240 |
| *Escherichia coli* | 2.3207 | 10.4660 | 7.50e-05 | 0.0016 |
| *Escherichia coli KTE103* | 1.6380 | 7.9032 | 0.0005 | 0.0061 |
| *Escherichia coli KTE71* | 1.7889 | 8.0445 | 0.0006 | 0.0068 |
| *Escherichia coli O104:H4 str Ec11 9990* | 1.3165 | 7.5988 | 0.0118 | 0.0467 |
| *Fibrobacter succinogenes subsp succinogenes S85* | -4.0054 | 11.2960 | 9.35e-05 | 0.0017 |
| *Fournierella massiliensis* | -1.7629 | 7.9835 | 0.0012 | 0.0120 |
| *Helicobacter canis* | 3.3398 | 9.8356 | 1.15e-05 | 0.0006 |
| *Helicobacter cf pullorum* | -3.0178 | 9.4195 | 0.0002 | 0.0024 |
| *Holdemania filiformis* | -1.4147 | 8.2255 | 0.0037 | 0.0216 |
| *Mycoplasma alvi ATCC 29626* | 1.6993 | 7.4913 | 0.0008 | 0.0090 |
| *Negativibacillus massiliensis* | -1.4487 | 8.6353 | 0.0074 | 0.0334 |
| *Phascolarctobacterium faecium* | -3.0704 | 8.7872 | 1.85e-05 | 0.0006 |
| *Roseburia hominis* | 2.0754 | 9.5271 | 0.0020 | 0.0155 |
| *Ruminococcus flavefaciens MC2020* | 2.4691 | 8.6355 | 0.0004 | 0.0054 |
| *Ruminococcus gnavus AGR2154* | -1.8559 | 7.9142 | 0.0018 | 0.0152 |
| *Ruminococcus lactaris CC59 002D* | -1.4291 | 7.9097 | 0.0067 | 0.0316 |
| *Ruminococcus torques* | -1.9140 | 9.2833 | 0.0025 | 0.0174 |
| *Sharpea azabuensis* | 2.3366 | 9.6263 | 0.0032 | 0.0191 |
| *Shigella sonnei* | 1.5177 | 8.1260 | 0.0026 | 0.0174 |
