# Supplementary material for: Escherichia coli is implicated in the development and manifestation of host susceptibility to the roundworm Trichostrongylus colubriformis infections in sheep
Source: Vet Res. 2025 Jul 1;56:133. doi: 10.1186/s13567-025-01565-1 (PMC12220768; doi:10.1186/s13567-025-01565-1)

**Additional file 4.** **Differential bacterial associations or interactions inferred using the NetCoMi algorithm**. The sign + means positive interactions. The sign – means negative interactions. The thickness of the edge (interaction line) indicates the strength of correlations. RES: the interaction or association network inferred from the resistant lambs. SUS: the association network inferred from susceptible lambs. The full species name can be found in Additional file 4. *N* = 20 per group.


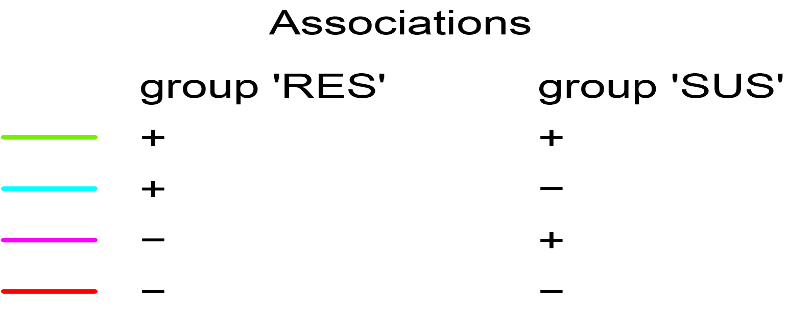

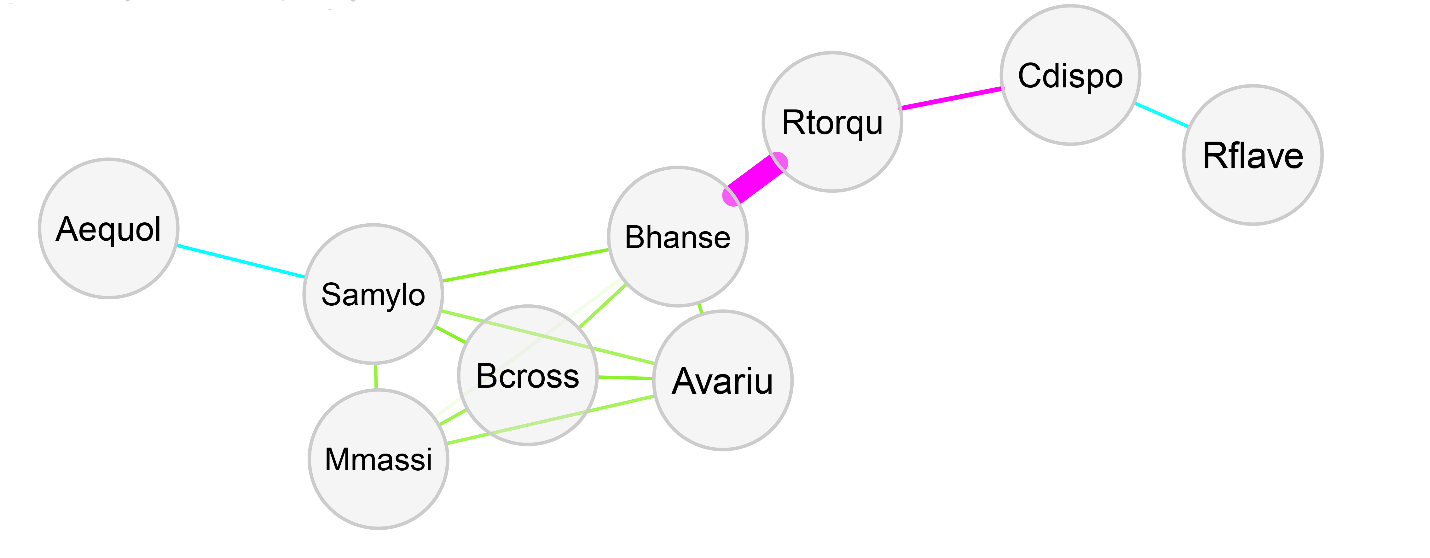

Supplement: Supplementary file 4 — Additional file 4.Differential bacterial associations or interactions inferred using the NetCoMi algorithm. The sign + means positive interactions. The sign – means negative interactions. The thickness of the edge (interaction line) indicates the strength of correlations. RES: the interaction or association network inferred from the resistant lambs. SUS: the association network inferred from susceptible lambs. The full species name can be found in Additional file 4. N = 20 per group. [file 13567_2025_1565_MOESM4_ESM.docx]
