# Supplementary material for: Escherichia coli is implicated in the development and manifestation of host susceptibility to the roundworm Trichostrongylus colubriformis infections in sheep
Source: Vet Res. 2025 Jul 1;56:133. doi: 10.1186/s13567-025-01565-1 (PMC12220768; doi:10.1186/s13567-025-01565-1)
Supplement: Supplementary file 5 — Additional file 5. Species abbreviations used for network inference and comparison. [file 13567_2025_1565_MOESM5_ESM.docx]

**Additional file 5** **Species abbreviations used for network inference and comparison.**

| Symbol | Scientific name |
| --- | --- |
| Aanaer | *Asteroleplasma anaerobium* |
| Aequol | *Adlercreutzia equolifaciens* |
| Aethan | *Acetivibrio ethanolgignens* |
| Aferme | *Acidaminococcus fermentans* |
| Afineg | *Alistipes finegoldii* |
| Ahadru | *Anaerostipes hadrus* |
| Ahista | *Allisonella histaminiformans* |
| Airreg | *Asaccharospora irregularis* |
| Alipol | *Anaerovibrio lipolyticus* |
| Amassi | *Alistipes massiliensis* |
| Amucin | *Akkermansia muciniphila* |
| Aputre | *Alistipes putredinis* |
| Arubii | *Anaerotruncus rubiinfantis* |
| Aseneg | *Alistipes senegalensis JC50* |
| Ashahi | *Alistipes shahii* |
| Asterc | *Anaerofustis stercorihominis* |
| Atimon | *Alistipes timonensis JC136* |
| Avariu | *Anaeroplasma varium* |
| Bacidi | *Bacteroides acidifaciens* |
| Balkai | *Bacillus alkalitolerans* |
| Bboum | *Bifidobacterium boum DSM 20432* |
| Bcacca | *Bacteroides caccae* |
| Bcopro | *Bacteroides coprocola* |
| Bcross | *Butyrivibrio crossotus* |
| Bfaeci | *Bacteroides faecichinchillae* |
| Bfaecs | *Butyricimonas faecihominis* |
| Bfineg | *Bacteroides finegoldii DSM 17565* |
| Bforam | *Bacillus foraminis* |
| Bfragi | *Bacteroides fragilis* |
| Bglobo | *Bifidobacterium globosum* |
| Bgluce | *Blautia glucerasea* |
| Bhanse | *Blautia hansenii* |
| Bhumi | *Bacillus humi* |
| Bhunga | *Butyrivibrio hungatei* |
| Binfan | *Bacillus infantis* |
| Blfaec | *Blautia faecis* |
| Bluti | *Blautia luti* |
| Bmasse | *Bacillus massiliogorillae* |
| Bmasss | *Blautia massiliensis* |
| Bmedit | *Bacillus mediterraneensis* |
| Bobeum | *Blautia obeum* |
| Boratu | *Bacteroides ovatus* |
| Bparav | *Butyricimonas paravirosa* |
| Bphoca | *Blautia phocaeensis* |
| Bprove | *Blautia provencensis* |
| Bpulli | *Butyricicoccus pullicaecorum* |
| Btesti | *Bacillus testis* |
| Btheta | *Bacteroides thetaiotaomicron* |
| Bunifo | *Bacteroides uniformis* |
| Bviros | *Butyricimonas virosa* |
| Bvulga | *Bacteroides vulgatus* |
| Bwexle | *Blautia wexlerae* |
| Bxylan | *Bacteroides xylanisolvens* |
| Cacnes | *Cutibacterium acnes* |
| Cauran | *Clostridium aurantibutyricum* |
| Cbeije | *Clostridium beijerinckii* |
| Cborni | *Clostridium bornimense* |
| Cbutyr | *Clostridium butyricum* |
| Ccatus | *Coprococcus catus* |
| Ccelat | *Clostridium celatum* |
| Cchart | *Clostridium chartatabidum* |
| Ccoli | *Campylobacter coli* |
| Ccomes | *Coprococcus comes* |
| Cdispo | *Clostridium disporicum* |
| Ciguan | *Campylobacter iguaniorum* |
| Cinnoc | *Clostridium innocuum* |
| Cjejun | *Campylobacter jejuni* |
| Clanie | *Campylobacter lanienae* |
| Clongi | *Clostridium longisporum* |
| Cmassi | *Colidextribacter massiliensis* |
| Cneona | *Clostridium neonatale* |
| Cparap | *Clostridium paraputrificum* |
| Cpolyn | *Clostridium polynesiense* |
| Croseu | *Clostridium roseum* |
| Crumin | *Cellulosilyticum ruminicola JCM 14822* |
| Csaccb | *Clostridium saccharoperbutylacetonicum* |
| Csaccm | *Clostridium saccharobutylicum DSM 13864* |
| Csaudi | *Clostridium saudiense* |
| Csubte | *Clostridium subterminale* |
| Cterti | *Clostridium tertium* |
| Cvince | *Clostridium vincentii* |
| Ddetox | *Denitrobacterium detoxificans* |
| Dformi | *Dorea formicigenerans* |
| Dlongi | *Dorea longicatena* |
| Dpiger | *Desulfovibrio piger* |
| Ealber | *Escherichia albertii* |
| Ebacte | *Erysipelotrichaceae bacterium GAM147* |
| Ecellu | *Eubacterium cellulosolvens 6* |
| Ecoli | *Escherichia coli* |
| Econto | *Eubacterium contortum* |
| Eelige | *Eubacterium eligens* |
| Ehalli | *Eubacterium hallii* |
| Elenta | *Eggerthella lenta* |
| Emassi | *Eisenbergiella massiliensis* |
| Emucos | *Enterorhabdus mucosicola DSM 19490* |
| Eramos | *Erysipelatoclostridium ramosum* |
| Esirae | *Eubacterium siraeum DSM 15702* |
| Etenue | *Eubacterium tenue* |
| Fcfpra | *Faecalibacterium cf. prausnitzii KLE1255* |
| Fcylin | *Faecalitalea cylindroides* |
| Fmassi | *Fournierella massiliensis* |
| Fnecro | *Fusobacterium necrophorum BFTR-1* |
| Fplaut | *Flavonifractor plautii* |
| Fpraus | *Faecalibacterium prausnitzii* |
| Fsacch | *Fusicatenibacter saccharivorans* |
| Fsucci | *Fibrobacter succinogenes* |
| Hapode | *Helicobacter apodemus* |
| Hcanis | *Helicobacter canis* |
| Hcfpu | *Helicobacter cf. pullorum* |
| Hcinae | *Helicobacter cinaedi* |
| Hfilif | *Holdemania filiformis* |
| Hhathe | *Hungatella hathewayi* |
| Hmassi | *Holdemania massiliensis AP2* |
| Ibartl | *Intestinibacter bartlettii* |
| Ibutyr | *Intestinimonas butyriciproducens* |
| Imassi | *Intestinimonas massiliensis* |
| Lamylo | *Lactobacillus amylovorus* |
| Lbacte | *Lachnospiraceae bacterium* |
| Lboron | *Lysinibacillus boronitolerans* |
| Lcleve | *Lawsonella clevelandensis* |
| Lcreso | *Lysinibacillus cresolivorans* |
| Lcrisp | *Lactobacillus crispatus* |
| Lferme | *Lactobacillus fermentum* |
| Lfusif | *Lysinibacillus fusiformis* |
| Llongo | *Lactonifactor longoviformis* |
| Llouem | *Lysinibacillus louembei* |
| Lmassi | *Lysinibacillus massiliensis* |
| Lmeyer | *Lysinibacillus meyeri* |
| Lmucos | *Lactobacillus mucosae* |
| Lodyss | *Lysinibacillus odysseyi* |
| Lpacae | *Lachnoclostridium pacaense* |
| Lpecti | *Lachnospira pectinoschiza* |
| Lsphae | *Lysinibacillus sphaericus* |
| Lurini | *Lachnoclostridium urinimassiliense* |
| Lxylan | *Lysinibacillus xylanilyticus* |
| Malvi | *Mycoplasma alvi ATCC 29626* |
| Mmassi | *Merdibacter massiliensis* |
| Mmoats | *Mycoplasma moatsii ATCC 27625* |
| Mpirum | *Mycoplasma pirum MPI25960* |
| Nmassi | *Negativibacillus massiliensis* |
| Ophoca | *Olsenella phocaeensis* |
| Orumin | *Oscillibacter ruminantium GH1* |
| Oscato | *Olsenella scatoligenes* |
| Osplan | *Odoribacter splanchnicus* |
| Oumbon | *Olsenella umbonata* |
| Ovaler | *Oscillibacter valericigenes* |
| Palben | *Prevotella albensis DSM 11370 = JCM 12258* |
| Pbifer | *Paraclostridium bifermentans* |
| Pbrevi | *Prevotella brevis* |
| Pbryan | *Prevotella bryantii* |
| Pcapil | *Pseudoflavonifractor capillosus* |
| Pdista | *Parabacteroides distasonis* |
| Pexcre | *Parasutterella excrementihominis* |
| Pfaeci | *Phascolarctobacterium faecium* |
| Prumia | *Prevotella ruminicola* |
| Prumin | *Pseudobutyrivibrio ruminis* |
| Psorde | *Paeniclostridium sordellii* |
| Psucci | *Phascolarctobacterium succinatutens YIT 12067* |
| Ralbus | *Ruminococcus albus* |
| Ramylo | *Ruminobacter amylophilus* |
| Rbromi | *Ruminococcus bromii* |
| Rcecic | *Roseburia cecicola* |
| Rfaeci | *Roseburia faecis* |
| Rflave | *Ruminococcus flavefaciens* |
| Rgnavu | *Ruminococcus gnavus* |
| Rhomia | *Romboutsia hominis* |
| Rhomin | *Roseburia hominis* |
| Rileal | *Romboutsia ilealis* |
| Rintes | *Roseburia intestinalis* |
| Rinuli | *Roseburia inulinivorans* |
| Rlacta | *Ruminococcus lactaris* |
| Rlitus | *Romboutsia lituseburensis* |
| Rmarit | *Romboutsia maritimum* |
| Rtimon | *Romboutsia timonensis* |
| Rtorqu | *Ruminococcus torques* |
| Samylo | *Succinimonas amylolytica* |
| Sazabu | *Sharpea azabuensis* |
| Sdextr | *Succinivibrio dextrinosolvens* |
| Sdysen | *Shigella dysenteriae 225-75* |
| Sequin | *Streptococcus equinus* |
| Sflexn | *Shigella flexneri SFJ17B* |
| Sgallo | *Streptococcus gallolyticus* |
| Sinfan | *Streptococcus infantarius* |
| Slutet | *Streptococcus lutetiensis* |
| Smassi | *Soleaferrea massiliensis* |
| Srumid | *Succiniclasticum ruminis DSM 9236* |
| Srumin | *Selenomonas ruminantium* |
| Ssilve | *Solibacillus silvestris* |
| Ssonne | *Shigella sonnei* |
| Ssucci | *Schwartzia succinivorans* |
| Ssucro | *Syntrophococcus sucromutans* |
| Stherm | *Streptococcus thermophilus TH985* |
| Svaria | *Subdoligranulum variabile* |
| Tbryan | *Treponema bryantii* |
| Tglyco | *Terrisporobacter glycolicus* |
| Tmayom | *Terrisporobacter mayombei* |
| Tnexil | *Tyzzerella nexilis* |
| Tothin | *Terrisporobacter othiniensis* |
| Trecta | *Treponema rectale* |
| Tsangu | *Turicibacter sanguinis* |
| Tziole | *Treponema zioleckii* |
